# An integrated analysis of spatial access to the three-tier healthcare delivery system in China: a case study of Hainan Island

**DOI:** 10.1186/s12939-021-01401-w

**Published:** 2021-02-12

**Authors:** Xiuli Wang, Barnabas C. Seyler, Wei Han, Jay Pan

**Affiliations:** 1 HEOA Group, West China School of Public Health and West China Fourth Hospital, Sichuan University, No.17 People’s South Road, Chengdu, 610041 China; 2 Institute for Healthy Cities and West China Research Center for Rural Health Development, Sichuan University, No.17 People’s South Road, Chengdu, China; 3 Department of Environment, Sichuan University, No.24 South Section 1, Yihuan Road, Chengdu, 610065 China; 4 Health, Nutrition and Population Global Practice, World Bank, No.1 Jianguomenwai Street, Chaoyang district, Beijing, 100020 China

**Keywords:** Three-tier health care delivery system, Spatial access, Enhanced two-step floating catchment area method (E2SFCA), Overlay analysis

## Abstract

**Background:**

Access to healthcare is critical for the implementation of Universal Health Coverage. With the development of healthcare insurance systems around the world, spatial impedance to healthcare institutions has attracted increasing attention. However, most spatial access methodologies have been developed in Western countries, whose healthcare systems are different from those in Low- and Middle-Income Countries (LMICs).

**Methods:**

Hainan Island was taken as an example to explore the utilization of modern spatial access techniques under China’s specialized Three-Tier Health Care Delivery System. Healthcare institutions were first classified according to the three tiers. Then shortest travel time was calculated for each institution’s tier, overlapped to identify eight types of multilevel healthcare access zones. Spatial access to doctors based on the Enhanced Two-Step Floating Catchment Area Method was also calculated.

**Results:**

On Hainan Island, about 90% of the population lived within a 60-min service range for Tier 3 (hospital) healthcare institutions, 80% lived within 30 min of Tier 2 (health centers), and 75% lived within 15 min of Tier 1 (clinics). Based on local policy, 76.36% of the population living in 48.52% of the area were able to receive timely services at all tiers of healthcare institutions. The weighted average access to doctors was 2.31 per thousand residents, but the regional disparity was large, with 64.66% being contributed by Tier 3 healthcare institutions.

**Conclusion:**

Spatial access to healthcare institutions on Hainan Island was generally good according to travel time and general abundance of doctors, but inequity between regions and imbalance between different healthcare institution tiers exist. Primary healthcare institutions, especially in Tier 2, should be strengthened.

**Supplementary Information:**

The online version contains supplementary material available at 10.1186/s12939-021-01401-w.

## Introduction

In 2005, the 58th World Health Assembly issued a call for Universal Health Coverage (UHC), which is defined as access to key promotive, preventive, curative, and rehabilitative health interventions for all at an affordable cost, thereby achieving equity in access [1, 2]. Now UHC has emerged as both a global and national health priority, and progressive realization of UHC is viewed as a critical path for improving health outcomes and achieving greater equity in health across all populations [3]. Therefore, detailed and accurate assessments of current healthcare systems are essential for future healthcare planning.

Access to healthcare is central to the performance of healthcare systems around the world [4], and variability in healthcare access has been identified as a main cause for inequality in health outcomes [5]. Healthcare access is defined by five specific dimensions: 1) availability, 2) accessibility, 3) accommodation, 4) affordability, and 5) acceptability [6]. Access can be further classified as either spatial or non-spatial, or potential versus revealed access [7]. The first two dimensions are spatial in nature and refer to the number of providers available and travel impedances in reaching them, while the latter three dimensions are essentially non-spatial and reflect healthcare financing arrangements and cultural factors. The fusion of availability and accessibility has been referred to as “spatial access”, which accounts for the spatial barriers that consumers must overcome to receive services [8]. Spatial access (e.g., dimensions 1–2) is also interpreted as potential access, which considers the possibility that people can access services, while revealed access depends on the willingness, preferences, and choices of individuals [9]. As an effective indicator, potential spatial access has been widely used in previous studies to measure access to healthcare and estimate equity [10].

With the improvement of healthcare insurance systems, spatial access to healthcare resources is attracting increasing attention [11, 12]. According to a survey conducted by China’s National Bureau of Statistics, during the past two decades, people’s financial access to healthcare has substantially improved, while spatial access to healthcare has become the primary reason why timely utilization of healthcare does not occur [13, 14]. Generally, three factors are critical when measuring spatial access to healthcare resources: 1) the service capacity of healthcare institutions, 2) population demand, and 3) geographic impedance [15]. Service capacity refers to the amount of resources each institution possesses, such as the number of beds, physicians, or other specific facilities [12, 16]. Population demand is the number of people who may need a particular service. Geographic impedance refers to the extent to which the distance between populations and service locations influence access [17], with geographic distance and travel time commonly utilized.

Various methods have been developed to estimate spatial access to healthcare resources, and they can be roughly classified into four categories: 1) travel impedance to nearest provider, 2) average travel impedance to a set of providers, 3) provider-to-population ratios (PPR), and 4) the gravity model [8]. Although combining measures of travel impedance and supply is necessary to properly understand spatial access [18], travel impedance to the nearest provider has been recognized as a good measure of spatial access in certain settings, particularly where provider choices are limited and nearest providers are the most likely to be used [8]. For example, nearest providers are the most commonly-used for emergency healthcare and blood transfusion services [19–21], by pregnant women and the poor [19, 20], as well as in rural areas and low- and middle-income countries (LMICs) [20, 22]. Average travel impedance to providers is calculated by summing up the travel impedance from every demand point to all providers, then averaged. This method is seldom utilized, because it over-weights the influence of providers located near the periphery of the study area [8]. PPR, which is the supply to demand ratio within administrative boundaries, is the most popular type of spatial access measure due to its ease of calculation. However, PPR does not reveal spatial variations within a boundary, and it does not account for interactions between supply and demand across boundaries [23]. The gravity model overcomes the shortcomings of PPR and is theoretically more robust, but it is also complex, requiring much more computation [23].

With the development of Geographical Information System (GIS) technologies, many new methods have been introduced based on the gravity model [24, 25]. Of these, the Two-Step Floating Catchment Area (2SFCA) method has garnered the most attention [25–27]. In the 2SFCA method, a catchment of reasonable radius (e.g., distance or travel time) centered on each supply location is generated, and the PPR within this catchment is calculated. Then, a catchment of reasonable radius centered on each demand location is generated, and the PPR of supply locations within these catchments are summed up to represent the spatial access of each demand location. However, the 2SFCA method has major limitations in that it assumes equal access within the catchment and no access outside the catchment is included in the analysis [28].

A number of improvements have been proposed based on the 2SFCA, and they can be classified into four types [9]. The first modifies the distance decay function, which models the likelihood trend that choosing a healthcare institution would decrease as the distance increases [29, 30]. The second strives to improve the definition of catchment areas [23, 31], while the third seeks to better account for the impacts of demand or supply side competition on access [17, 32, 33]. The fourth type of improvement attempts to incorporate assumptions concerning travel behaviors of service users [34, 35]. However, a major limitation in current application is that most spatial access methodologies have been developed in Western countries, which have very different healthcare systems than developing countries. In China, the disparity of size between administrative units (usually counties) is quite large, and remote areas with limited healthcare resources are normally much larger than populous areas. Moreover, cross-boundary health seeking behaviors are very common in China, so although PPR is the most commonly used spatial access measure in Chinese government reports [36], it is not the most suitable measure.

China’s healthcare system is characterized by a Three-Tier Health Care Delivery System (Table 1) [37–40]. Based on government policy, the highest tier (Tier 3) includes county hospitals in rural regions and city hospitals in urban regions, which are responsible for most inpatient services as well as teaching and research missions. All hospitals must be operated under the supervision of certain government departments to ensure their legality. The mid-level tier (Tier 2) includes township health centers (THCs, 乡镇卫生院, *xiāngzhèn wèishēng yuàn*, in Chinese) in rural regions and community health centers/stations (CHCs) in urban regions. In certain communities where the service area or population is too large for its community health center (社区卫生服务中心, *shèqū wèishēng fúwù zhōngxīn*) to handle, a community health station (社区卫生服务站, *shèqū wèishēng fúwù zhàn*) is established subordinate to the community health center to augment its service capacity. THCs and CHCs have responsibility for maintaining the public health status of their respective communities, providing public health services, preventing and treating common health problems, managing chronic diseases, and providing rehabilitation services. The front-line tier (Tier 1) institutions include village clinics (村卫生室, *cūn wèishēng shì*) in rural areas and community clinics (诊所, *zhěnsuǒ*) in urban areas, and their functions include health education, prevention, promotion, and home visits [36]. THCs (Tier 2) and village clinics (Tier 1) in rural areas, as well as CHCs (Tier 2) in urban areas, are funded and partially operated by local governments, so apart from providing basic clinical care, they are also responsible for many public health services [40]. Urban community clinics (Tier 1) on the other hand, are largely driven by market demands, usually being opened by registered (assistant) doctors. But, due to their having more experienced healthcare professionals (in contrast to village clinics, which tend to have less-rigorously qualified village doctors) also provide much of the primary healthcare services in urban areas [26].

**Table 1:** The Three-Tier Healthcare Delivery System in China

Tier	Healthcare institution		Function based on national documents	Local policy in Hainan Province
	Rural area	Urban area		
Tier 3	County Hospitals	City Hospitals	Diagnose and treat common and frequently-occurring diseases; Provide emergency treatment of emergency/severe diseases and referrals for complicated diseases; Provide training and guidance to primary healthcare institution personnel; Provide relevant public health services and emergency medical treatment/first aid.	**Plan on healthcare service system of Hainan Province (2015–2020) (Document number 2015** [53]**)** By the year 2020, set up five regional medical centers in the east, west, south, north, and center of Hainan Island, and construct healthcare institutions in populous areas, such as Wenchang, Wanning, and Dongfang, for the purpose of achieving “one-hour circle to hospital care”, and to treat the majority of diseases within island.
Tier 2	Township Health Centers	Community Health Centers/Stations	Provide basic public health services such as disease prevention and control; Offer options for treatment, nursing, and rehabilitation of common health problems within the community; Undertake management of public health services; Provide management and technical guidance of village clinics; Provide training of village doctors.	**Action plan for standardized construction of primary healthcare institutions in Hainan Province (Document number 2018** [54]**)** Focus on constructing a “30-min to primary care in rural areas and 15-min to primary care in urban areas” healthcare system, to ensure safe, effective, economical, and convenient healthcare services for both urban and rural residents, to push forward the healthcare reform targets of “minor diseases not requiring travel to cities and major diseases not requiring island departure”.
Tier 1	Village Clinics	Clinics	Provide basic public health services; Provide primary treatment of ordinary, common, and frequently occurring diseases in the village/community.	

Note: The function of the three tiers of healthcare institutions was depicted according to the State Council’s National Healthcare Policy-announcement of strategic plan on National Health Care Service System (2015–2020)

Spatial access research in China has been increasing in recent years (see Appendices 1–2), apart from assessing healthcare resource allocation in different study areas (usually at the county or metropolis-level such as in Beijing or Shenzhen, but with a few analyses at the province level) [41–44], modifications on the 2SFCA method have been made to better fit the reality in China. The most common modification has been the utilization of online navigation systems based on actual travel preferences within China (e.g., public transportation, private car, etc.), which allow for real time travel impedance to be calculated to get more accurate spatial access estimates [45, 46]. Second, which tiers and/or types of healthcare institutions being considered depend on individual research questions. In most cases, the catchment area sizes of different tiers/levels/types of healthcare institutions are set differently, and general access is calculated as the sum of access for all healthcare institutions [9, 47]. However, Zhang et al. [48] measured shortest travel time to city-level hospitals, county-level hospitals, and community-level health centers, identifying different types of healthcare resource shortage areas. However, that study’s categorizations of healthcare institutions did not strictly follow government-mandated categories, and the definitions of “under-served areas” were based on patient perceptions (via interview data) without considering government guidelines or benchmarks. Existing literature provides concise indicators to express the general spatial access of healthcare resources. However, the incorporation of policy outcomes (e.g., from government or other authoritative bodies, intended to focus healthcare resource priorities) into academic studies to assess the effectives of healthcare practices has thus far been insufficient, making it difficult for governments to assess the effectiveness and weaknesses of current approaches.

In China, government guidance and investment play the central role in the healthcare system. Thus, this study sought to take Hainan Island (a relatively closed-loop location within China) as a case study to assess spatial access to healthcare resources from the perspective of policy makers, and thereby provide guidance for future planning and policy making in broader contexts within China and other LMICs. Specifically, we sought to: 1) explore the utility of existing spatial access assessment methods under China’s specialized Three-Tier Health Care Delivery System; 2) precisely assess the spatial access to healthcare resources on Hainan Island based on local policies; and 3) use Hainan’s example to propose recommendations for future healthcare planning to policy makers.

## Methods

### Study area

**Fig. 1:**
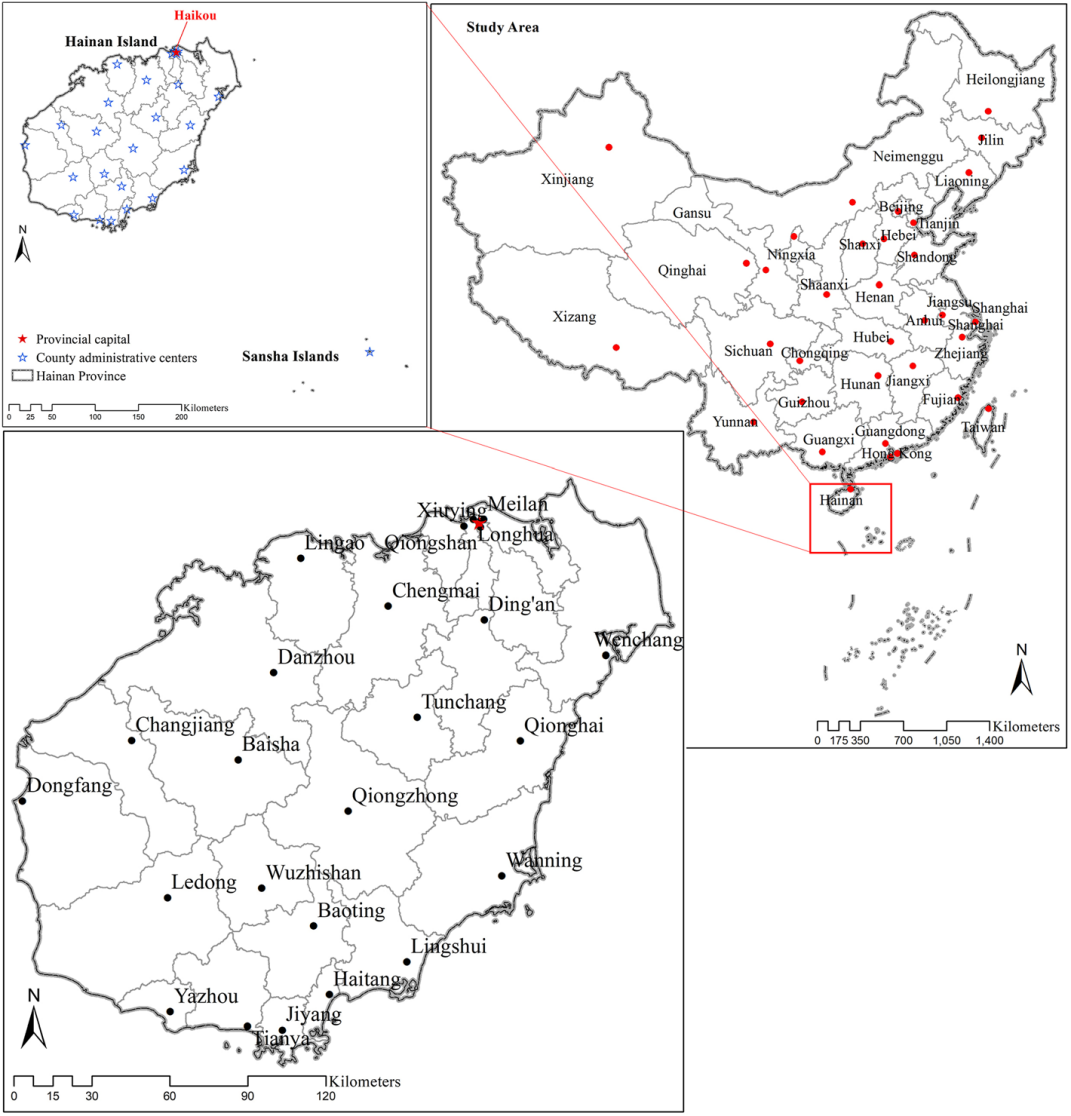
Study area

Hainan Island, located just off the southern coast of Mainland China, is the largest populated island located entirely within the South China Sea. Hainan was selected for this study because, as an island, the cross-boundary health seeking behaviors of patients is relatively limited compared with other areas in China. Hainan Island, together with Sansha Islands, and the surrounding sea areas in the South China Sea, form Hainan Province (Fig. 1). However, almost all of Hainan Province’s population is concentrated on Hainan Island. The total land area of Hainan Province is 35,191 km^2^, but Sansha Islands account for just 788 km^2^. The total population (permanent population in 2018) is 9.34 million, while just over 500 permanent residents live in Sansha Islands [49].

Transportation between Hainan Island and Mainland China relies on ships and airplanes. Ships traveling from Hai’an Harbor in Guangdong Province to Haikou City on the north end of Hainan Island take between 2 and 3 h. There are also two airports on Hainan Island, Meilan Airport in Haikou City (north end) and Fenghuang Airport in Sanya City (south end).

**Fig. 2:**
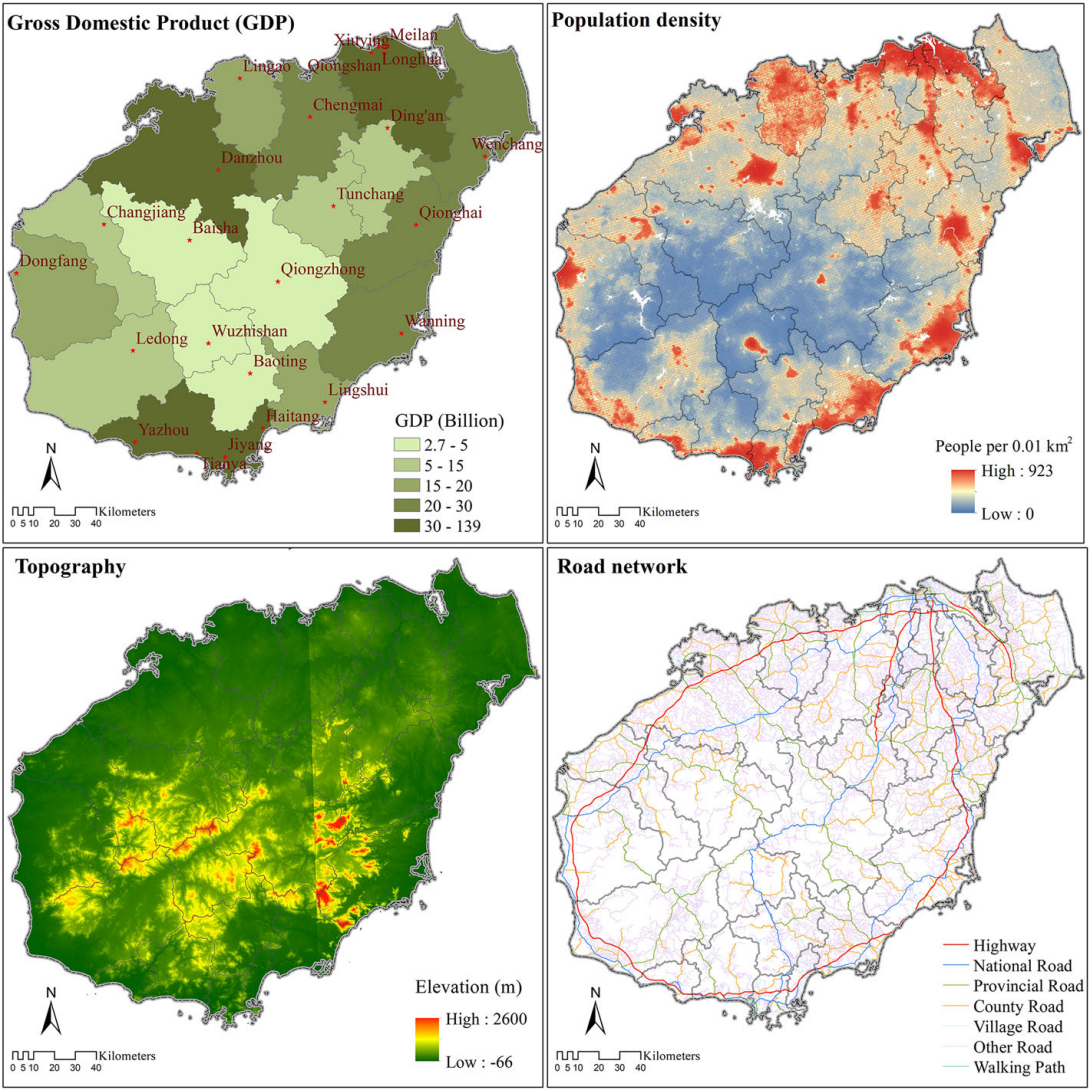
Population, topography, transportation, and economic development of Hainan Island, China. Note: Population density is calculated by using the WorldPop 2015 dataset and population data extracted from *Hainan Statistical Yearbook 2019*. The road network data, administrative boundary data, and elevation data were collected from the National Geomatics Center of China. GDP data was collected from *Hainan Statistical Yearbook 2019* (reporting data from 2018)

On Hainan Island, the coastal areas are more developed than the central region, with the island’s population also distributed more densely along the coast. The road networks along the coasts are more developed, and the counties/districts there also have higher regional GDP. Overall, the central part of the island has the lowest population density, is the most mountainous, and is the least developed (Fig. 2).

### Data sources

On the supply side, healthcare institution data were extracted from the yearly report of the Health Commission of Hainan Province, including Tier 3 hospitals; Tier 2 THCs and CHCs; and Tier 1 village clinics and community clinics. Community clinics were included as the first tier of the healthcare system in urban areas because community clinics provide significant numbers of basic clinical care in urban areas and include considerable numbers of registered (assistant) doctors. For example, in 2018, there were 6.12 million outpatient visits to urban community clinics in Hainan Province, compared with 7.05 million to rural village clinics. There were 3022 registered (assistant) doctors in community clinics in Hainan Province (2018), compared with just 589 in village clinics [49]. For each type of healthcare institution, we acquired basic data (e.g., name, address, type) and service capacity data (e.g., number of registered doctors and registered assistant doctors).

On the demand side, population data were collected from both WorldPop, which provides an estimate of population per grid (100*100 m) with national totals adjusted to match UN population division estimates from 2010, 2015, and 2020 [50], and the *Hainan Statistical Yearbook 2019*, which provides permanent population data at each county/district in 2018 [49]. The administrative boundary data, elevation data, and road network data were downloaded from the National Geomatics Center of China [51].

### Data pretreatment

First, all healthcare institutional data were classified according to the three healthcare system tiers. For each tier of healthcare institutions, the number of doctors (including registered doctors and registered assistant doctors) was utilized as service capacity indicators in this project. Based on the definition listed in the Health Statistics Yearbook of China 2018 [36], registered doctors and registered assistant doctors are health professionals possessing Medical Practitioner’s Licenses qualifying them to carry out medical treatment and preventative healthcare, which means they are the only authorized healthcare professionals that can offer these services.

Second, population density data were adjusted based on the population data by county/district from the statistical yearbook and gridded population data from WorldPop 2015 [48]. Permanent population data from the statistical yearbook was regarded as real population data and was allocated to 100*100 m^2^ grids according to the value per cell of the WorldPop dataset, with the following formula:

1 $$ {P}_{ij}={G}_{ij}\ast \left(\frac{T_j}{G_j}\right) $$

Where *P*_*ij*_ is the corrected population density value of grid i in the county/district j, *G*_*ij*_ is the corresponding WorldPop value of population density. *G*_*j*_ is the sum of the grid values for WorldPop in county/district j, and *T*_*j*_ is the real population in the county/district j based on the *Hainan Statistical Yearbook 2019*.

Third, a geo-database containing suppliers (healthcare institutions), demanders (population density expressed as raster pixels), and geographical impedances (travel time based on road network) was established. For healthcare institutions, their longitude and latitude coordinates were compiled based on their addresses in Baidu Map, then geocoded as points in GIS. The service capacity indicators (e.g., number of doctors) were set as attributes of the points. For population density, the precision of population density data in the second step was adjusted from 100*100 m^2^ to 1*1 km^2^ by summing grid populations due to computation limitations, and every raster pixel represented a single resident point (e.g., indicating demand). The road network was classified by both type and attribute of speed class. Type of roads reveals the distribution of different roads on Hainan Island, but the travel speed of each section of road was influenced by several additional factors including road type, landscape, road width, materials, and so on. Thus, speed limits for each road section were assigned based on speed class, with the fastest being 90 km/h and the slowest being 5 km/h. Travel time based on the road network was utilized as geographic impedance of patients receiving healthcare services.

### Spatial access assessment methodology

In order to propose recommendations for future health planning to local policy makers, we assessed spatial access on Hainan Island based on local policies, including the “one-hour circle to hospital care” [52, 53] and “30-minutes to primary care in rural areas and 15-minutes to primary care in urban areas” [53, 54] policies proposed by the Hainan Provincial Government. We also used the standard made by the State Council’s National Healthcare Policy (2015–2020) that by 2020 there should be 2.50 registered (assistant) doctors for every thousand people [40]. Thus, an overlap analysis based on the Nearest-Neighbor Method, and the Enhanced Two Step Floating Catchment Area Method was utilized to assess spatial access.

### The nearest-neighbor method

For the first step, the Nearest-Neighbor Method was utilized to calculate the shortest travel time for resident points (e.g., demand location). For each resident point, only the nearest healthcare institution (e.g., supply location) was considered when using the nearest-neighbor method. The travel impedance between the demand location and the supply location represented the convenience of receiving healthcare services. Shorter travel impedance indicates that patients can receive more timely treatment [26].

Based on the Hainan Provincial Government’s “one-hour circle to hospital care” and “30-minutes to primary care in rural areas and 15-minutes to primary care in urban areas” policies, the shortest travel time to the three tiers of healthcare institutions were classified into 6 categories: 0–15, 15–30, 30–60, 60–90, 90–120, and > 120 min. Driving time along the road network represented travel impedance, and the service area command under network analysis tools in ArcGIS 10.2 was utilized to calculate the results.

### Overlap analysis

On the second step, an overlap analysis was utilized to identify different types of healthcare institution access zones (Table 2). Still based on the “one-hour circle to hospital care” and “30-minutes to primary care in rural areas and 15-minutes to primary care in urban areas” policies, timely access to healthcare institutions was defined as less than 60 min from the nearest hospital (Tier 3 healthcare institution), less than 30 min from the nearest primary healthcare institution (Tier 2 and Tier 1) in rural areas (e.g., THCs and village clinics), and less than 15 min in urban areas (e.g., CHCs and community clinics).

**Table 2:** Identification of under-served areas in terms of travel time

Type	Multilevel healthcare access zones
A	Effective service zone
B	Lack of Tier 1 healthcare facilities
C	Lack of Tier 2 healthcare facilities
D	Lack of Tier 3 healthcare facilities
E	Lack of Tier 2 and Tier 1 healthcare facilities
F	Lack of Tier 3 and Tier 1 healthcare facilities
G	Lack of Tier 3 and Tier 2 healthcare facilities
H	Lack of all tiers of healthcare facilities

Note: The Tier 1 healthcare institutions are clinics in urban areas and village clinics in rural areas. The Tier 2 healthcare institutions are CHCs in urban areas and THCs in rural areas. The Tier 3 healthcare institutions are hospitals. Based on the “one-hour circle to hospital care” and “30-min to primary care in rural areas and 15-min to primary care in urban areas” policy of Hainan Province, areas that do not meet the following requirements were identified as under-served:

Tier 3 healthcare institutions: travel time to hospital ≤60 min;

Tier 2 healthcare institutions: travel time to THCs ≤30 min or to CHCs ≤15 min;

Tier 1 healthcare institutions: travel time to village clinic ≤30 min or to urban clinic ≤15 min

### The enhanced two step floating catchment area method

On the third step, the Enhanced Two Step Floating Catchment Area (E2SFCA) Method was utilized to calculate spatial access to doctors from the three tiers of healthcare institutions. Developed to overcome the limitation of the 2SFCA Method which assumes equal accessibility within catchments and no accessibility outside catchments, the E2SFCA Method divides each catchment into multiple sub-catchments and applies different weights for each sub-catchment to model the distance decay effect [30]. The weights are defined by a weight function which can be adjusted depending on the type or importance of a service/resource [28]. The E2SFCA method has been used in a variety of studies in different regions of the world including China [15, 26, 41, 55–57]. However, previous studies have only used this method to calculate access to healthcare resources generally, but in our study we calculated access separately for each tier of healthcare institutions in order more rigorously ascertain how effectively healthcare policies (e.g., benchmarks) are being reached.

The E2SFCA Method can be represented as follows:

2 $$ {R}_j=\frac{S_j}{\sum_{i\in \left({d}_{ij}\in {D}_r\right)}{P}_k{W}_r} $$

3 $$ {A}_i^F={\sum}_{j\in \left({d}_{ij}\in {D}_r\right)}{R}_j{W}_r $$

The first equation calculates the supply-to-demand ratio of each healthcare institution j. A healthcare resource (e.g., doctors in this study) of a healthcare institution j (*S*_*j*_ in eq. 1) is shared by the population within the reachable range (*P*_*k*_ in eq. 1), but the accessibility decreases as the reachable range (e.g., the catchment area, *D*_*r*_ in Eq. [1], where *d*_*kj*_ is the travel time between resident point k and institution j) gets further from the center (e.g., the supply point j). Distance-weights (*W*_*r*_ in Eq. [1]), derived from a distance decay function represent the decreasing trend.

The second equation calculates the spatial access of each resident point i (${\mathrm{A}}_i^F$ in eq. 2). The healthcare institutions that are closer to the resident point are considered more important and have higher distance-weights (W_*r*_).

The E2SFCA method was implemented in ArcGIS 10.2. Spatial access to doctors for the three healthcare institution tiers were calculated separately. To be consistent with the Nearest-Neighbor Method, three catchment areas (0–15 min, 15–30 min, 30–60 min) for each healthcare institution and resident point were generated, with weights being 0.880, 0.316, and 0.01 respectively [12, 26]. The weights were calculated using Gaussian function with β being 440. Weighted averages for spatial access of every county/district were calculated using SPSS 20.0, and Gini Coefficient of spatial access to doctors was calculated under StataSE 12 environment to measure inequality on Hainan Island [58].

## Results

### General distribution of healthcare institutions on Hainan Island

**Fig. 3:**
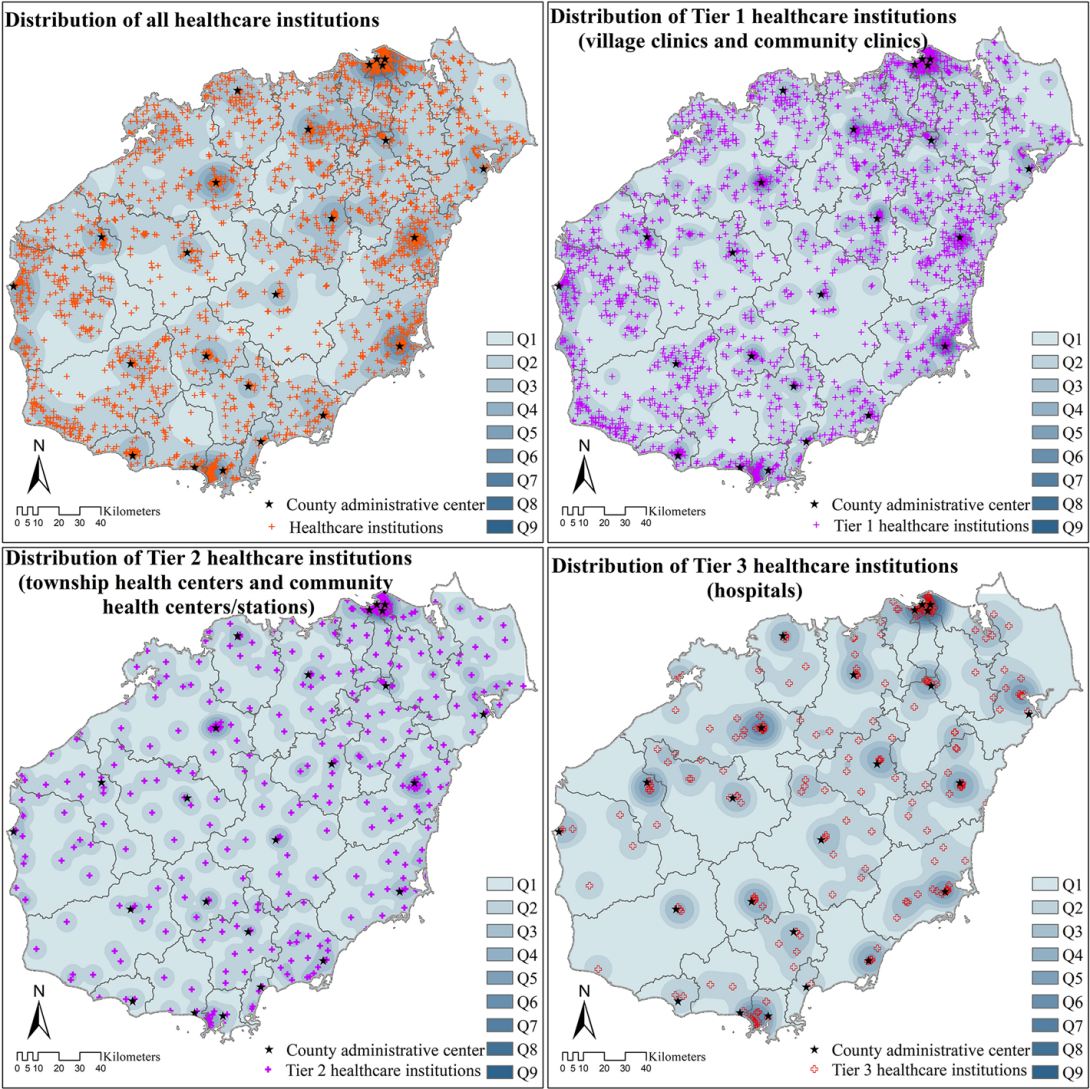
Distribution of healthcare institutions on Hainan Island (2018). Note: Q1-Q9 represent the cluster degree of healthcare institutions based on Kernel Density Analysis in ArcGIS 10.2, higher numbers indicate higher cluster degree

In 2018, there were 255 hospitals, 177 CHCs, 299 THCs, 1787 clinics, and 2716 village clinics on Hainan Island. The healthcare institutions were widely distributed across Hainan Island. Tier 3 healthcare institutions (hospitals) were often located near the administrative centers of each district/county, but mostly concentrated in Haikou City. Tier 2 healthcare institutions (CHCs in urban areas and THCs in rural areas) also clustered near the administrative centers, but were more evenly distributed compared to Tier 3 healthcare institutions. Nevertheless, the most significant cluster of Tier 2 healthcare institutions was also located in Haikou. The distribution of Tier 1 healthcare institutions (community clinics in urban areas and village clinics in rural areas) was most even, although Haikou still had the most significant cluster (Fig. 3).

### Shortest travel time to healthcare institutions

**Fig. 4:**
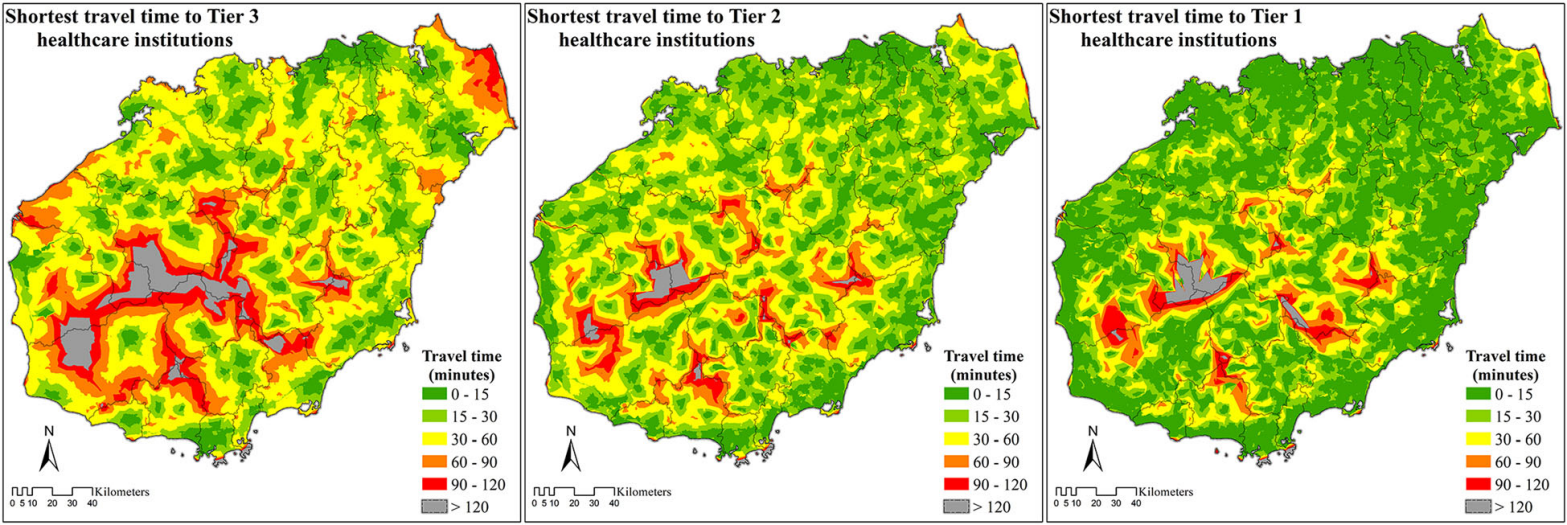
Shortest travel time to the three tiers of healthcare institutions. Note: The Tier 1 healthcare institutions are clinics in urban areas and village clinics in rural areas. The Tier 2 healthcare institutions are CHCs in urban areas and THCs in rural areas. The Tier 3 healthcare institutions are hospitals

On Hainan Island, areas further than 1 h from the nearest healthcare institution were all concentrated in the central mountainous region. The northeast part of Hainan Island generally showed better access to healthcare institutions than the southwest (Fig. 4).

In total, about 10% of the population (0.93 million) lived further than 1 h from the nearest Tier 3 healthcare institution, and 0.91% of the population (0.08 million) lived further than 2 h from the nearest Tier 3 healthcare institution. Only 3.48% (0.32 million) and 2.19% (0.20 million) of the population lived further than 1 h from Tier 2 and Tier 1 healthcare institutions, respectively, and only about 0.3% of the population lived further than 2 h from any healthcare institution (Table 3).

**Table 3:** Populations covered by the six travel zones of the three tiers of healthcare institutions

Travel zone	Tier 1	Tier 2	Tier 3
0–15 Minutes	7,000,677 (75.33%)	4,701,942 (50.59%)	3,442,280 (37.04%)
15–30 Minutes	1,503,997 (16.18%)	2,809,747 (30.23%)	2,254,765 (24.26%)
30–60 Minutes	585,316 (6.30%)	1,458,979 (15.70%)	2,665,765 (28.68%)
60–90 Minutes	124,836 (1.34%)	230,756 (2.48%)	652,004 (7.02%)
90–120 Minutes	49,765 (0.54%)	66,590 (0.72%)	194,815 (2.10%)
> 120 Minutes	29,260 (0.31%)	25,836 (0.28%)	84,223 (0.91%)
Total	9,293,851 (100%)	9,293,851 (100%)	9,293,851 (100%)

Note: Tier 1 healthcare institutions are clinics in urban areas and village clinics in rural areas. Tier 2 healthcare institutions are CHCs in urban areas and THCs in rural areas. Tier 3 healthcare institutions are hospitals

In total 75.33% of the population lived within 15 min from a Tier 1 healthcare institution, and more than 90% lived within 30 min. For Tier 2 healthcare institutions, about 50% of the population lived within a 15-min service area, and another 30% lived within a 30-min service area. For Tier 3 healthcare institutions, about 37% of the population lived within 15 min of the nearest institution, accounting for 3.44 million people, the population living within the 30–60 min service area (28.68%) was slightly more than within the 15–30 min service area (24.26%) (Table 3).

### Overlap analysis

**Fig. 5:**
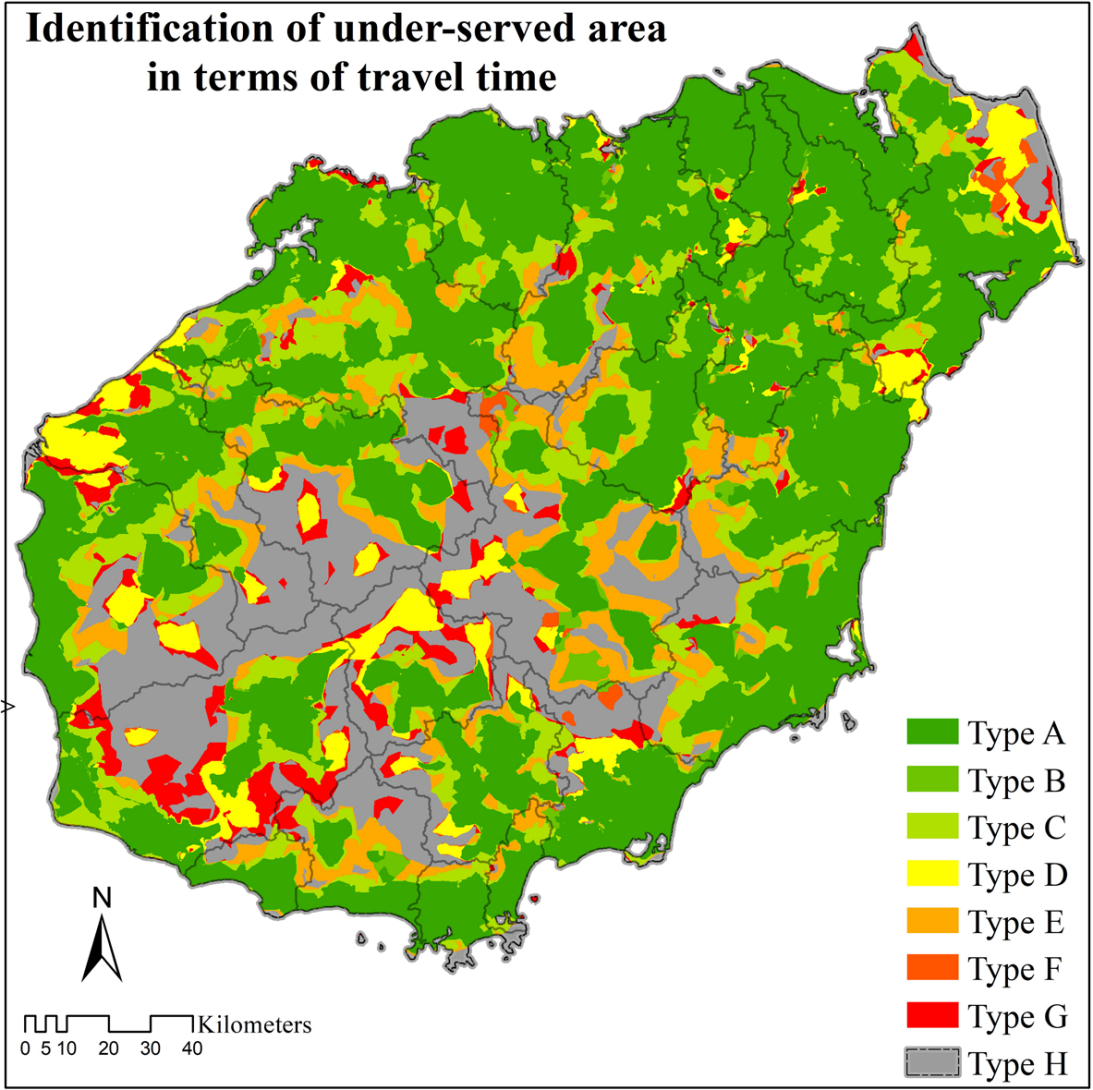
Overlap analysis of shortest travel time to the three healthcare institution tiers. Note: Type A indicates coverage by all three Tiers of healthcare institutions; Type B indicates coverage by both Tier 3 and Tier 2 healthcare institutions; Type C indicates coverage by both Tier 3 and Tier 1 healthcare institutions; Type D indicates coverage by both Tier 2 and Tier 1 healthcare institutions; Type E indicates coverage by only Tier 3 healthcare institutions; Type F indicates coverage by only Tier 2 healthcare institutions; Type G indicates coverage by only Tier 1 healthcare institutions; Type H indicates no coverage by any healthcare institutions

On Hainan Island, more than 76% of the population (living in about 49% of Hainan Island’s area) lived in Type A: effective service zone (i.e., able to receive timely service at all three tiers of healthcare institutions). Only about 5% of the population (living in 18% of the island’s area) cannot receive timely service from any of the three tiers of healthcare institutions (Type H). The second highest percentage of the population fell within Type C coverage, meaning they cannot get timely Tier 2 healthcare service (Table 4). Under-served populations were mostly located in the southwest and center of Hainan Island (Fig. 5).

**Table 4:** Percentage of area and population covered within the eight types of under-served areas in terms of travel time on Hainan Island (2018)

Type	Multilevel healthcare access zones	Area %	Population %
A	Effective service zone	48.52%	76.36%
B	Lack of Tier 1 healthcare facilities	1.02%	0.53%
C	Lack of Tier 2 healthcare facilities	13.33%	8.81%
D	Lack of Tier 3 healthcare facilities	5.16%	2.80%
E	Lack of Tier 2 and Tier 1 healthcare facilities	8.61%	3.86%
F	Lack of Tier 3 and Tier 1 healthcare facilities	0.67%	0.30%
G	Lack of Tier 3 and Tier 2 healthcare facilities	4.43%	2.11%
H	Lack of all tiers of healthcare facilities	18.27%	5.22%

Note: Tier 1 healthcare institutions are clinics in urban areas and village clinics in rural areas. Tier 2 healthcare institutions are CHCs in urban areas and THCs in rural areas. Tier 3 healthcare institutions are hospitals

### Spatial access to doctors

**Fig. 6:**
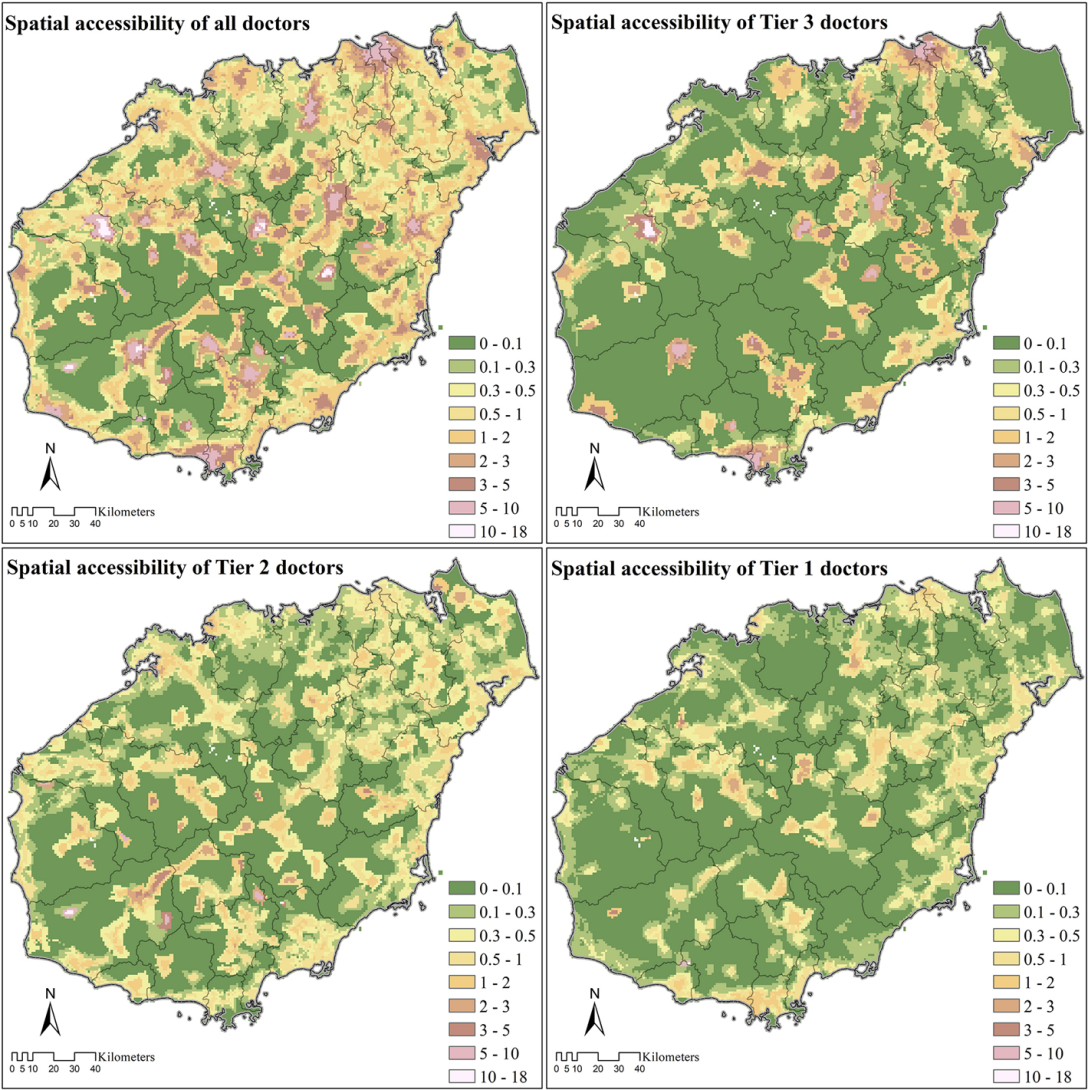
Spatial access to doctors on Hainan Island. Note: The Tier 1 healthcare institutions are clinics in urban areas and village clinics in rural areas. The Tier 2 healthcare institutions are CHCs in urban areas and THCs in rural areas. The Tier 3 healthcare institutions are hospitals

Spatial access to doctors was not equally distributed on Hainan Island, with hotspots located near administrative centers of each county. In general, spatial access to doctors was more even and widespread for Tier 2 healthcare institutions and more evenly distributed on the northeast part of Hainan Island (Fig. 6).

The population weighted average of spatial access to doctors was 2.31 per thousand on Hainan Island, among which 64.66% (1.50) were contributed by Tier 3 healthcare institutions, 18.56% (0.43) were contributed by Tier 2 healthcare institutions, and the rest 16.78% (0.39) were contributed by Tier 1 healthcare institutions.

The Gini Coefficient of spatial access to doctors was 0.56 on Hainan Island. In contrast with the standard set by the State Council’s National Healthcare Policy (2015–2020) [40], which was 2.50 doctors per thousand population by 2020, only 32.19% of the population living in 9.21% of the area of Hainan Island met the requirement. Compared to developed countries such as Japan, the United Kingdom, and the United States, the percent population on Hainan Island reaching the average number of doctors per thousand in those countries was similarly limited (Table 5).

**Table 5:** Percentage of population and area on Hainan Island with access to doctors exceeding national and international standards (2018)

Country	Doctors per thousand population	Area	Population
Japan	2.41 (2016)	9.70%	32.84%
China	2.5 (2020)	9.21%	32.19%
UK	2.81 (2017)	7.84%	30.14%
USA	2.59 (2016)	8.78%	31.58%

Note: The standard of China was extracted from the National healthcare policy announcement of strategic plan on National healthcare service system (2015–2020), while the standards from Japan, UK, and USA were extracted from the Global Health Observatory (GHO) data

## Discussions

Hainan Island is isolated from Mainland China with limited connection to healthcare resources outside of the island, thus it can be considered as a relatively closed-loop system for healthcare policy analysis and resource allocation. Additionally, compared with the economically advanced regions along China’s eastern and southern coasts, such as Shanghai and Shenzhen, which have carried out many previous regional reforms, the Hainan Government mostly follows China’s Central Government directives from Beijing. Thus, the experiences from Hainan Island have great applicability far beyond Hainan itself.

### Convenience of receiving healthcare services on Hainan Island

The Hainan Provincial Government proposed the “one-hour circle to hospital care” and the “30-minutes to primary care in rural areas and 15-minutes to primary care in urban areas” policies, and, based on these standards, our results show that spatial access to healthcare institutions on Hainan Island is generally effective. Of the 9.26 million people living on Hainan Island, 89.98% can access to Tier 3 healthcare institutions within 1 h, 80.82% can access to Tier 2 healthcare institutions within 30 min (compared to 50.59% within 15 min), and 91.51% can access to Tier 1 healthcare institutions within 30 min (compared to 75.33% within 15 min). Thus, regardless of urbanization level, the spatial goals for Tier 3 and Tier 1 healthcare facilities have been roughly achieved. There remains, however, a relatively sizable gap for Tier 2 access.

Furthermore, the overlap analysis results show that 76.36% of Hainan’s total population and 48.52% of its total area fulfilled the policy goals for timely access to healthcare. Of the different access zone types, the lack of timely Tier 2 service on Hainan Island was most significant. The underserved access zones of Type C, E, G, and H, which relate to the lack of timely access to Tier 2, involved 20.00% of the total population and 44.64% of Hainan’s total area.

In the Three-Tier Health Care Delivery System, Tier 2 healthcare facilities are intended to function as the central hubs between Tier 3 and Tier 1 healthcare institutions [26, 38]. Although patients in China increasingly choose to access higher level hospitals [59], the Tier 2 facilities still play important roles in the healthcare system, especially in rural areas. For example, in 2018 in Hainan, the total number of outpatient visits was 50.79 million person-times, among which 39.5% were to hospitals, while 54.3% were to primary healthcare institutions. In addition, of the 27.59 million outpatient visits to primary healthcare institutions, CHCs accounted for 10.2% and THCs accounted for 41.3%. The total number of inpatient visits was 1.19 million, with 86.2% occurring in hospitals and only 7.7% occurring in primary healthcare (Tier 1–2) institutions. Amongst the 0.09 million inpatient visits in primary healthcare institutions, 10.8% occurred in CHCs, while 86.1% occurred in THCs [49]. Also, apart from basic clinical care, as primary healthcare institutions funded by the government, THCs and village clinics in rural areas, as well as CHCs in urban areas, are responsible for many public health services [60]. For example, they offer basic public health service packages providing 14 individual services, including health record keeping, health management of certain types of vulnerable populations, health education programs, vaccinations, etc. A primary intended purpose for Tier 2 healthcare institutions in China is to relieve pressure on Tier 3 institutions. However, on Hainan Island, greater focus should be made to improve spatial coverage for Tier 2 healthcare institutions since their coverage is currently inadequate by government standards.

On Hainan Island, areas that did not meet the government guidelines were generally concentrated in the central mountainous region. As a tourism location, visitors usually choose to go to the coastal areas, which also tend to attract large numbers of young migrant worker populations. Consequently, the central mountainous regions tend to be populated by older permanent residents. Considering the relatively less developed road network and lower GDP in the central island, residents in these under-served areas require more attention from the local government.

### Abundance of healthcare resources on Hainan Island

On Hainan Island in 2018, the weighted average of spatial access to doctors was 2.31 per thousand people, which was slightly lower than the standard set by the State Council’s National Healthcare Policy (2015–2020) (2.50 doctors/1000 people) [40]. It was also lower than the spatial access to doctors in some developed countries like Japan (2.41 doctors/1000), the United Kingdom (2.81 doctors/1000), and the United States (2.59 doctors/1000, Table 5) [61]. But unlike general practitioners which function as gatekeepers (e.g., providing referrals) to medical specialists in developed countries, health professionals in China’s primary healthcare institutions tend to possess lower levels of academic degrees, technical titles, and licenses compared with health professionals working in hospitals [62]. For example, in 2017, only 30.1% of registered (assistant) doctors in hospitals lacked a university degree (graduating instead from technical high schools with medical certifications or technical colleges with associate medical degrees), however, the corresponding numbers were much higher in lower tiered healthcare institutions, including CHCs (55.2%), THCs (82.5%), and village clinics (97.7%) [36]. So even though the absolute gap in spatial access was limited compared with developed countries, the actual differences based on medical training and knowhow is likely much greater.

The absolute total number gap in spatial access to doctors was relatively limited on Hainan Island, but the share of doctors represented by primary healthcare institutions (Tier 1 and Tier 2) seems to be problematic. On Hainan Island only 35.44% of the access to doctors was provided by these primary healthcare institutions. Under the framework of the tiered healthcare delivery system (分级诊疗, *fēnjí zhěnliáo*, in Chinese) [26, 63], which is an idealized situation envisioned for healthcare services in China [5, 60, 62], primary healthcare institutions should provide certain key services: 1) Basic public health services such as preventative care, health education, and family planning; 2) Clinical treatment for common and frequently-occurring diseases; 3) Rehabilitation and nursing services for certain diseases; and 4) Referral of patients when demand exceeds service capacity or knowhow [40]. The limited percentage of registered (assistant) doctors in primary healthcare institutions on Hainan Island did not meet the tiered health-care delivery system policy guidelines.

We also found that inequality in geographical distribution of doctor resources was significant on Hainan Island. Although the weighted average of spatial access to doctors (2.31 doctors/1000 people) was close to the national guidelines (2.5 doctors/1000), less than 1/3 of Hainan’s population actually enjoyed spatial access to doctors that met the guidelines (Table 5). Most of those who did were distributed in the more developed populous urban and coastal areas. This situation could be improved by increasing the percentage of healthcare resources in the lower tiers of healthcare institutions.

## Conclusions

Hainan Island’s population is generally well-covered by hospital care (Tier 3), but access to primary healthcare institutions (Tier 2 and Tier 1) still requires improvement, especially for Tier 2 healthcare institutions. Although access to doctors is close to national guidelines, as well as the standards of many developed countries, doctors in Hainan are unequally distributed geographically, and their distribution is not equitable across the different healthcare institution tiers.

Based on the current allocation of healthcare resources on Hainan Island, and aiming to achieve the provincial government’s goals of timely access to healthcare and “Receiving Treatment at the Local Level” policy [54], we recommend that Hainan’s government seek to increase the service capacity of primary healthcare institutions, especially for THCs and CHCs, which are regarded as central hubs in the Three-Tier Health Care System. First, more Tier 2 healthcare institutions should be constructed to enlarge their service coverage area. Second, more healthcare resources, especially well-trained doctors should be allocated to primary healthcare institutions to improve their service capacities and quality. Third, a healthcare quality assessment system for primary healthcare institutions should be developed to monitor their operation.

Several limitations of this analysis should also be considered. First, we utilized driving time along roads to represent geographical impedance without considering the possibility of other transportation modes. Also, transportation modes might be different in urban and rural areas, while targeting different tiers of healthcare institutions [64]. Second, the distance decay function utilized in the E2SFCA method was based on previous research in other Chinese provinces, but for more accuracy future studies should adapt these for the current reality on Hainan Island. The catchment sizes might also be different for different healthcare institutions based on their tier and service capacities. Thirdly, the demand for healthcare services was considered only to population size while different socio-economic groups (age, sex, wealth, etc.) have diverse requirements [64]. As a tourism site, the seasonal tourists and migrant workers should also be considered in future healthcare resource allocation studies on Hainan Island.

## Supplementary Information

**Additional file 1.** Literature review of spatial access to healthcare related research in China.

**Additional file 2.** Spatial access to healthcare publications in China over time.

## Data Availability

Part of the data that support the findings of this study are available from the Health Commission of Hainan Province, but restrictions apply to the availability of these data, which were used under license for the current study, and so are not publicly available. Data are, however, available from the authors upon reasonable request and with permission of the Health Commission of Hainan Province. Part of the data analyzed during the current study are available from the corresponding author on reasonable request.

## Abbreviations

UHC: Universal Health Coverage
LMICs: Low- and Middle-Income Countries
E2SFCA: Enhanced Two-Step Floating Catchment Area
PPR: Provider-to-population ratios
GIS: Geographical Information System
2SFCA: Two-Step Floating Catchment Area
THCs: Township health centers
CHCs: Community health centers
