# Supplementary figures and images for: An integrated analysis of spatial access to the three-tier healthcare delivery system in China: a case study of Hainan Island
Source: Int J Equity Health. 2021 Feb 12;20:60. doi: 10.1186/s12939-021-01401-w (PMC7881625; doi:10.1186/s12939-021-01401-w)

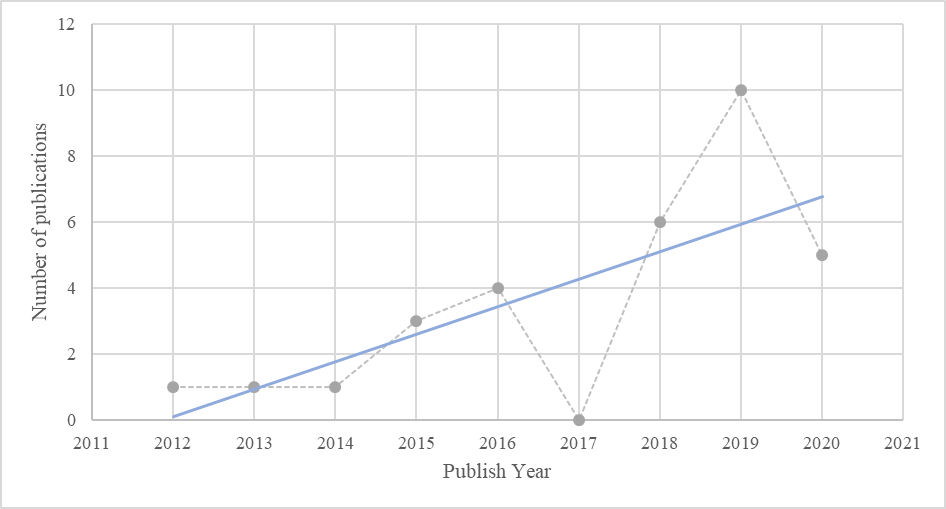


**Appendix 2**. Spatial access to healthcare publications in China over time

Supplement: Supplementary file 2 — Additional file 2. Spatial access to healthcare publications in China over time. [file 12939_2021_1401_MOESM2_ESM.docx]
